# PCSK9 Monoclonal Antibodies: New Developments and Their Relevance in a Nucleic Acid–Based Therapy Era

**DOI:** 10.1007/s11883-022-01053-3

**Published:** 2022-07-28

**Authors:** Ioanna Gouni-Berthold, Jonas Schwarz, Heiner K. Berthold

**Affiliations:** 1 Center for Endocrinology, Diabetes and Preventive Medicine, University of Cologne, Faculty of Medicine and University Hospital Cologne, Kerpener Str. 62, 50937 Cologne, Germany; 2 Department of Internal Medicine and Geriatrics, Bethel Clinic (EvKB) and University Hospital OWL, Campus Bielefeld-Bethel, Bielefeld, Germany

**Keywords:** PCSK9 antibodies, Evolocumab, Alirocumab, siRNA, Antisense oligonucleotide, CRISPR

## Abstract

**Purpose of Review:**

To report on recent data about PCSK9 monoclonal antibodies and to evaluate their relevance in a nucleic acid–based therapy era for lipid lowering and prevention of cardiovascular disease.

**Recent Findings:**

New methods of *PCSK9* inhibition based on nucleic acid therapeutics such as antisense oligonucleotides, small interfering RNAs, and CRISPR tools for therapeutic gene editing are reported, and interesting new data regarding the clinical relevance of PCSK9 antibodies are discussed.

**Summary:**

Promising methods of *PCSK9* inhibition are in development, and one of them, the siRNA inclisiran targeting *PCSK9*, has already been approved for clinical use. However, PCSK9-mAb remains the PCSK9-inhibiting tool with the longest safety data and the only one having positive cardiovascular outcome trials. An ongoing cardiovascular outcome trial with inclisiran is planned to be completed in 2026. Other forms of PCSK9 inhibition, such as antisense oligonucleotides targeting *PCSK9* and CRISPR base editing of *PCSK9*, are still in early phases of development, and their potential clinical relevance remains to be established.

## Introduction

Hypercholesterolemia is a causal risk factor for atherosclerotic vascular disease (ASCVD), and ASCVD remains the leading cause of death worldwide [1, 2]. Proprotein convertase subtilisin-kexin type 9 (PCSK9) plays a major role in cholesterol homeostasis by decreasing the amount of low-density lipoprotein (LDL) receptors on plasma membranes, thus increasing the serum levels of LDL cholesterol (LDL-C) [3]. PCSK9 monoclonal antibodies (PCSK9-mAb) decrease LDL-C by inhibiting circulating PCSK9 and therefore promoting the recycling of the LDL receptor in the cell surface. The currently available PCSK9-mAb, evolocumab and alirocumab, have been shown to not only robustly decrease LDL-C by 50–60% on top of statins but also to decrease the risk of cardiovascular events with a very good safety profile [4, 5]. During the last couple of years, new methods of inhibiting PCSK9 have been under development, namely, nucleic acid–based therapies. They represent an entirely new way to inhibit or “silence” the expression of *PCSK*9. These agents include N-acetylgalactosamine (GalNAc)-conjugated antisense oligonucleotides (ASO) and small interfering RNAs (siRNA), and new gene-editing options such as clustered regularly interspaced short palindromic repeats (CRISPR) tools or meganucleases delivered by adeno-associated virus (AAV) vectors. The siRNA targeting *PCSK9* inclisiran has already been approved by both the FDA and EMA, for the treatment of hypercholesterolemia in USA and Europe, respectively.

In their totality, these new treatment options may significantly enlarge our armamentarium of lipid-lowering medications if proven to be safe and to decrease cardiovascular events. In the present review, we will discuss the new nucleic acid–based therapies to inhibit *PCSK9* as well as recent data on PCSK9-mAb, which we consider of interest.

## Nucleic Acid–Based Therapies and PCSK9 Inhibition

**Fig. 1:**
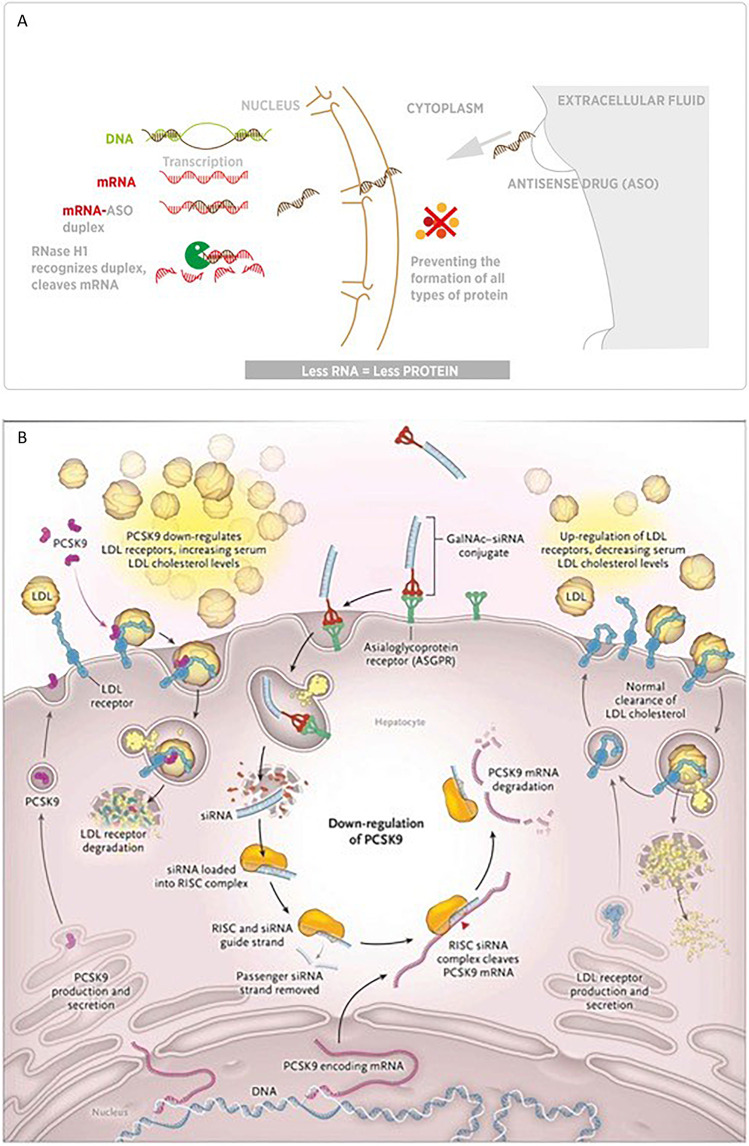

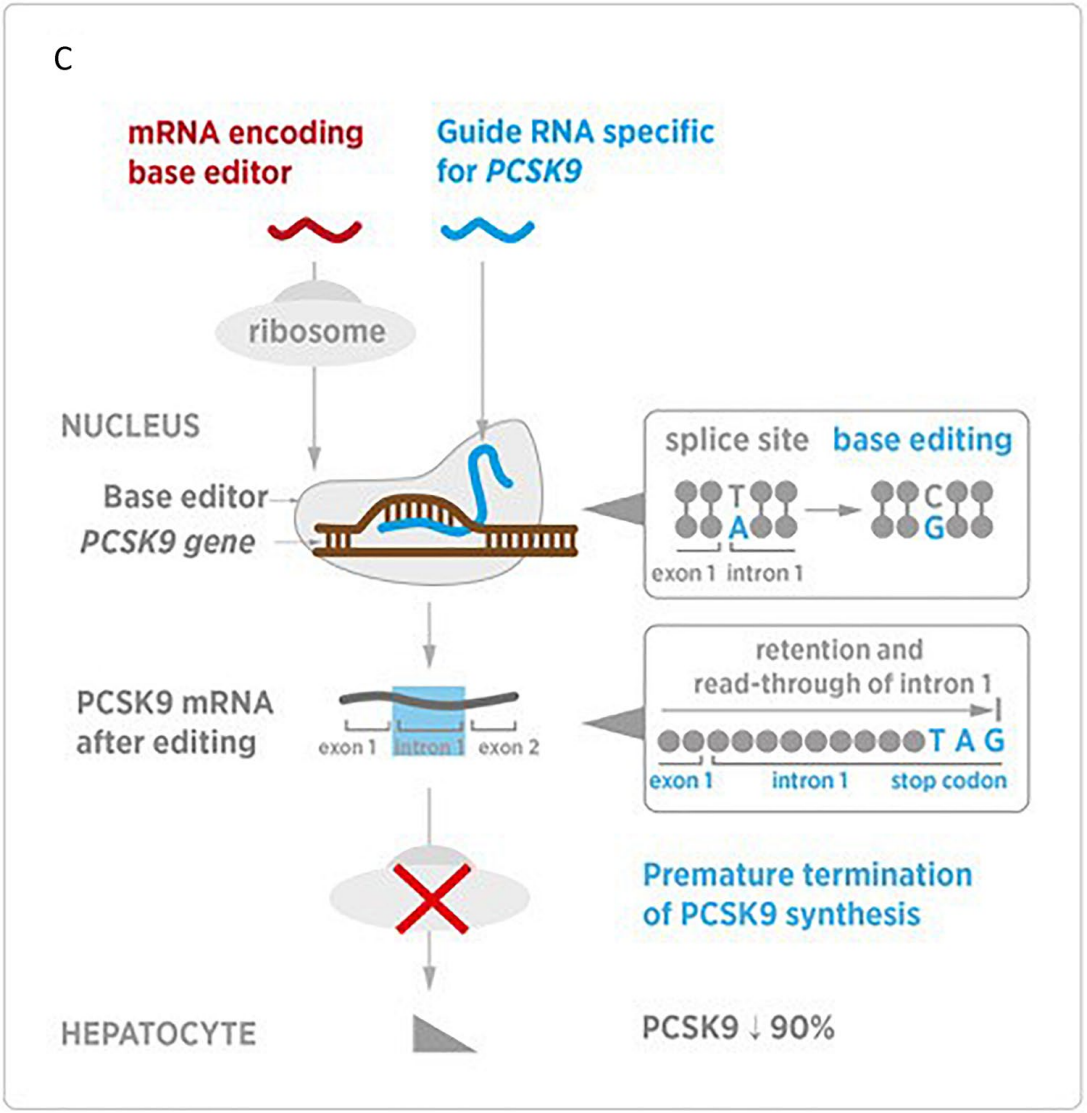
Mechanism of action of nucleic acid-based therapies. **A** ASO, **B** siRNA inclisiran, **C** genome editing of *PCSK9* using CRISPR base editors (**A**: modified from Tsimikas et al., with permission [66]; **B**: modified from Warden BA and Duell PB, with permission [7•]; **C**: modified from Katzmann et al. [20••].)

Nucleic acid–based therapies represent an entirely new way to inhibit or “silence” the expression of *PCSK9*, decrease LDL-C, and potentially prevent the development of ASCVD. They include ligand-modified ASOs (Fig. 1A) and small siRNAs (Fig. 1B) and gene editing tools such as CRISPR base editing (Fig. 1C) or meganucleases [6]. ASOs are single-stranded modified DNA molecules, and siRNAs are double-stranded RNA molecules. Typical siRNA agents consist of a guide strand and a passenger strand with 21–23 nucleotides per strand, while typical ASOs consist of a single strand of about 20 nucleotides [7•]. ASOs and siRNA are conjugated with an N-acetylgalactosamine (GalNAc) moiety to improve liver-specific delivery and help reduce off-target effects. The GalNAc moiety represents a significant improvement over earlier versions of siRNA and ASO therapies because it allows lower drug doses and thus fewer side effects such as injection site reactions, hepatotoxicity, and thrombocytopenia. The GalNAc moiety directs the drug to the hepatocyte by binding to the asialoglycoprotein receptor (ASGPR), which is expressed almost exclusively in the liver. Then the siRNA or ASO enters the hepatocyte via receptor-mediated endocytosis, where due to the acidic pH the GalNAc-siRNA/ASO conjugate dissociates from the ASGPR, which recycles back to the cell surface.

siRNA enters the cytoplasm and the guide strand, which contains a complementary sequence to the target mRNA, and recruits and binds several proteins that constitute the RNA-induced silencing complex (RISC). The passenger strand, whose function is to stabilize the guide strand and to facilitate the RISC formation, then dissociates from the guide strand and is degraded (Fig. 1B) [7•]. The guide strand (bound to the RISC complex) binds a PCSK9 mRNA molecule and targets it for RISC-mediated degradation. While the plasma half-life of siRNAs is short (about 1 h), the functional half-life in the hepatocyte spans several weeks and can degrade multiple PCSK9 mRNA molecules [8, 9•].

ASOs entering the cytoplasm and the nucleus bind the complementary mRNA with Watson–Crick hybridization and activate the cleavage of the mRNA strand by the single-stranded RNase H (which functions both in the nucleus and in the cytosol) [10]. Different from the mode of action of siRNAs, the ASO-induced mRNA cleavage has a one-to-one stoichiometry [8].

CRISPR adenine base editors (ABE) have been recently used for in vivo editing of *PCSK9* [11••, 12•]. The two major studies in non-human primates reporting this method used an ABE (adenine deaminase linked to a catalytically impaired Cas9), which is guided to target a specific adenosine for chemical modification and induces a single-strand nick in the opposite strand. The A-to-G edit (T-to-C edit on the opposing strand) resulting from nick repair can be exploited to disrupt canonical splice sites. Together with a single-guide RNA (gRNA) targeting a *PCSK9* splice site, the ABE-encoding mRNA is encased in a lipid nanoparticle for delivery to the targeted liver cells. In both studies, the gRNA was designed to target the GT splice donor site at the boundary of *PCSK9* exon 1 and intron 1, converting it into GC. This approach leads to read-through into intron 1 and generates an in-frame stop codon.

### Small Interfering RNA–Targeting PCSK9

A siRNA-targeting *PCSK9,* inclisiran, is already approved by both FDA and EMA and is in clinical use in the USA and Europe for lowering LDL-C levels in patients with (i) primary hypercholesterolemia (familial or not) or mixed dyslipidemia in combination with a statin or with a statin and another lipid-lowering agent in patients that are not at their LDL-C target or (ii) alone or in combination with other lipid-lowering drugs (without a statin) in patients with statin intolerance or in those where a statin is contraindicated.

The dosage of inclisiran is 284 mg in 1.5 ml volume to be given subcutaneously (SC) at the beginning of treatment, after 3 months, and thereafter every 6 months.

In order to reduce susceptibility to degradation of inclisiran by endo- and exonucleases, it has been modified with phosphorothioate substitutions and the inclusion of 20-deoxy, 20-O-methyl, and 20-fluoro nucleotides. Peak levels occur 4 h after SC administration, and plasma concentrations cannot be detected after 48 h since it is rapidly and selectively taken up by the liver. It is catabolized by nucleases to shorter inactive nucleotides. Inclisiran has the benefit which is not inhibiting or inducing common drug transporters or cytochrome P450 enzymes; therefore, it is not expected to have any drug–drug interactions. After a single injection, the onset of PCSK9 and LDL-C lowering in plasma is first observed at day 14, with maximal effects between days 30 and 60. The plasma half-life of inclisiran ranges between 5 and 10 h, but the duration of PCSK9 and LDL-C lowering is prolonged, with an efficacy half-life of about 300 days [7•]. Renal clearance is its main route of elimination, and hemodialysis should not be performed for at least 72 h after inclisiran dosing. No dose adjustment is necessary in patients with mild, moderate, or severe renal impairment or in patients with mild-to-moderate hepatic impairment (there are no studies available on patients with severe hepatic impairment).

Inclisiran has two large clinical development programs called ORION and VICTORION [13, 14]. Till now, the study results show a consistent LDL-C reduction of about 50% and an 80% decrease in circulating PCSK9 levels [15•]. The data available till now suggest that inclisiran is an effective, safe, and well-tolerated treatment to lower LDL-C [15•]. The most common side effect seems to be injection site reactions. Bronchitis was more common in the inclisiran group compared with placebo in ORION 10 (patients with ASCVD) [16] and numerically but not statistically significant in ORION-9 (patients with heterozygous familial hypercholesterolemia [HeFH]) [17] and ORION-11 (patients with ASCVD or ASCVD risk equivalents) [16]. However, since the longest duration of studies with inclisiran was 18 months, treatment safety for longer periods of time requires further investigation. The cardiovascular endpoint trial with inclisiran, ORION-4, with 15,000 participants with ASCVD is currently recruiting and is expected to be completed in 2026 [18].

### Antisense Oligonucleotides Targeting PCSK9

Gennemark et al. developed a highly potent 16-nucleotide ASO (AZD8233) targeting *PCSK9* which can potentially be also used orally, unlike the majority of ASOs which are given only SC [19••]. As mentioned above, it has a GalNAc conjugation that provides liver specificity. In a single-blind phase 1 study of 73 healthy male subjects with LDL-C levels ≥ 100 mg/dl, a single SC dose of 90 mg AZD8233 reduced PCSK9 by up to 95% and LDL-C by 68%. These values slowly returned to baseline over 16 weeks after dosing. The trial was not powered, however, for evaluation of efficacy or the degree of reduction in PCSK9 or lipid biomarkers since it was planned to assess the safety, tolerability, pharmacokinetics, and pharmacodynamics of AZD8233.

In order to make this ASO active via oral administration, it was co-formulated in a tablet with sodium caprate as permeation enhancer. Repeated oral daily dosing in dogs resulted in a bioavailability of 7% in the targeted organ, the liver. A 14-day study in cynomolgus monkeys showed that all oral doses used (28, 42, and 56 mg/day) were associated with a decrease in PCSK9 of > 50% and a decrease in LDL-C levels between 45 and 50%. Considering that the half-life of the drug is 18 days, it is likely that this is an underestimation of the LDL-C-lowering effect. In all cases, the AZD8233 tablets were well tolerated except for transient instances of liquid feces.

Mathematical modeling suggested that a dose of 25 mg SC once a month or a daily oral dose of 15 mg is predicted to reduce circulating PCSK9 levels by approximately 80% at steady state, making AZD8233 a promising compound for further development. Phase 2 trials with the SC formulation of AZD8233 are ongoing. Trials in humans with the oral formulation are pending.

### PCSK9 Editing Using Meganucleases

Meganucleases, engineered from endonucleases, introduce double-strand breaks at specific sequences of a gene [20••]. Wang et al. showed in 2018 in non-human primates (*Rhesus macaques*) that single infusions of an AAV vector expressing an engineered meganuclease targeting *PCSK9* resulted in a dose-dependent decrease in circulating PCSK9 and serum LDL-C levels of up to 84% and 60%, respectively [21]. There was stable *PCSK9* editing of up to 46%. Transient asymptomatic elevations of serum transaminases due to a T-cell response against the transgene product were observed a few weeks after treatment and lasted for a few additional weeks to months. Vector DNA and meganuclease expression declined rapidly, while there were stable populations of genome-edited hepatocytes. There were approximately 30 off-target cleavage sites in the liver.

A second-generation *PCSK9*-specific meganuclease was developed, which showed less off-target cleavage. After 3 years of follow-up, the frequency of off-target editing continued but remained stable and no obvious adverse changes in the histopathology of the liver were detected [22••]. The reduction in circulating PCSK9 and LDL-C levels was also sustained over the 3 years of follow-up. A recent study showed that modification of the AAV vector to reduce the expression of the second-generation meganuclease eliminates much of the off-target editing in treated non-human primates, but with some potential attenuation of *PCSK9* editing [23]. Whether this method of gene editing will be further developed remains to be seen since off-target editing; the potential large degree of integration of the AAV vector sequence into the genome at the site of the break and the immune response are significant disadvantages.

### CRISPR Base Editing of PCSK9

Musunuru et al., as mentioned previously, used CRISPR adenine base editors (ABE) which inactivate genes by disrupting splice donors or splice acceptors at exon–intron boundaries [11••]. They were able to show that the CRISPR ABE used, ABE8.8, one of the latest ABEs, delivered along with the PCSK9 gRNA in vivo to living cynomolgus monkeys *(Macaca fascicularis*) via a single infusion of lipid nanoparticles (LNP) formulated to contain ABE8.8 mRNA and PCSK9 gRNA at a 1:1 ratio (0.5 mg/kg, 1 mg/kg, and 1.5 mg/kg), can induce near-complete knockdown of *PCSK9* with parallel reductions in circulating levels of PCSK9 and LDL-C of about 90% and 60%, respectively. These reductions occurred 2 weeks after the injection and remained stable for at least 8 months. Over 60% base editing was achieved in the liver. Since about 70% of the liver consists of hepatocytes, a 70% editing means DNA editing in virtually all hepatocytes. A rate of 60% editing is quite satisfactory.

There were immediate mild to moderate elevations of serum transaminases, most likely due to the lipid nanoparticle, which were transient and resolved in 1–2 weeks. There was no off-target editing when assessing the human genome and low-level (< 1%) at only one site in the monkey genome. The potential benefit of the CRISPR base editing is that, if it proves to be safe and effective in human studies, a one-time administration can be functional for decades compared to the twice a year administration of inclisiran or the every 2- or 4-week administration of the PCSK9-mAb.

In the same year (2021), Rothgangl et al. also examined the efficacy and safety of ABEs in the livers of mice and cynomolgus macaques for the reduction of PCSK9- and LDL-C levels [12•]. The ABE ABEmax was used to knock out *PCSK9* by introducing a splice-site mutation. An mRNA encoding ABEmax together with a chemically modified gRNA targeting the GT splice donor site of *PCSK9* intron 1 was co-formulated (1:1 ratio) in lipid nanoparticles and injected intravenously (dose 0.75 mg/kg or 1.5 mg/kg). There was an up to 67% editing (average 61%) in mice and up to 34% editing (average 26%) in macaques. Plasma PCSK9- and LDL-C levels were stably decreased by 95% and 58% in mice and by 32% and 14% in macaques, respectively. There were four groups tested (low dose or high dose, as either a single dose or two doses with a 2-week interval between dosing). The base editing frequency was 2% for the low dose and 28% for the high dose. Animals receiving a single high-dose infusion showed a 26% decrease in circulating PCSK9 and a 9% decrease in serum LDL-C 29 days after drug administration, while those who received two high-dose infusions had a 39% decrease in PCSK9 and an LDL-C decrease of 19% after 29 days. Re-dosing did not increase editing.

Immune responses such as evidenced by elevated levels of serum transaminases, pro-inflammatory cytokines, and immune-modulating chemokines were mild, transient, and most likely caused by the lipid nanoparticle formulation. In addition, *Sp*Cas9- and TadA-specific immunoglobulin G (IgG) antibodies were detected in monkeys that received two doses, which may explain the rather modest effect of the second dose. ABE mRNA was rapidly cleared with no off-target mutations in genomic DNA observed.

While the results of Musunuru et al. [11••] showed greater editing efficiency than those by Rothgangl et al. [12•], this could be related to the different versions of ABE used, dosing schedules (the effect of repeated dosing might have been diminished by immune responses), and length of study (8 months vs. 29 days).

Both works reported rapid clearance of the base-editor RNA and of the lipid nanoparticles. Advantages of nanoparticles over AAV vectors is their transient delivery, while AAV vectors can cause permanent integration into the genome or long-lasting expression leading to greater off-target effects and stronger immune responses [24]. While lipid nanoparticles also seem to induce immune responses, a potential option to decrease their intensity could be the incorporation of a GalNac molecule, which would enhance their liver specificity [25]. Still, concerns remain regarding whether base editing of *PCSK9* is a safe method for the treatment of hypercholesterolemia. Even small potential off-target effects will be permanent, and, as mentioned above, there is a potential for immunogenicity [26].

A summary of the various nucleic acid–based methods of *PCSK9* inhibition compared to PCSK9-mAb is shown in Table 1. While nucleic acid–based methods of *PCSK9* inhibition may be future therapy options, the PCSK9 antibodies are well established in clinical use. In the second part of this review, we will discuss recent developments with these drugs.

**Table 1:** Summary of LDL-C- and PCSK9-lowering effects (% change from baseline) of PCSK9-mAb and nucleic acid–based approaches for PCSK9 inhibition

Species	Mechanism of action (drug)	Decrease of circulating PCSK9 levels	Decrease of LDL-C levels	References
Human	PCSK9-mAb (alirocumab, evolocumab)	~ 90–100%	~ 50–60%	[4, 5, 56–60, 67]
Human	siRNA (inclisiran)	~ 80%	~ 50%	[7•, 15•, 16, 17, 68]
Human	ASO (AZD8233)	Up to ~ 95%	~ 70%*	[19••]
NHP	PCSK9 editing using a meganuclease	Up to ~ 85%	Up to ~ 60%	[21, 22••]
NHP	PCSK9 editing using base editor (ABE8.8)	~ 90%	~ 60%	[11••]
NHP	PCSK9 editing using base editor (ABEmax)	Up to ~ 40%	Up to ~ 20%	[12•]

*PCSK9-mAb* proprotein convertase subtilisin–kexin type 9 monoclonal antibodies, *siRNA* small interfering RNAs, *ASO* antisense oligonucleotides, *ABE* adenine base editors, *NHP* non-human primates

^*^Study not powered for evaluation of degree of reduction in PCSK9 levels or in lipid biomarkers

## Recent Clinical Data on PCSK9-mAb

### Morbidity and Mortality

Guedeney et al. performed a review of randomized controlled trials (RCTs) up to March 2018 comparing treatment with the PCSK9-mAb alirocumab or evolocumab vs. placebo or other lipid-lowering therapies [27•]. Primary efficacy endpoints were all-cause death, cardiovascular death, myocardial infarction (MI), and stroke. Thirty-nine RCTs comprising 66,478 patients of whom 14,639 were treated with alirocumab, 21,257 were treated with evolocumab, and 30,582 controls were included. The mean follow-up of the trials was 2.3 years with a total exposure time of 150,617 patient-years. Overall, the effects of PCSK9 inhibition on all-cause death and cardiovascular death were not statistically significant (1.03 vs. 1.15 per 100 patient-years; RR 0.89, 95% CI 0.75–1.04; *P* = 0.15 and 0.66 vs. 0.73 per 100 patient-years; RR 0.94, 95% CI 0.84–1.06; *P* = 0.34, respectively). However, PCSK9-mAb use was associated with a 20% lower risk of myocardial infarction (MI), a 22% lower risk of ischemic stroke, and a 17% lower risk of coronary revascularization (all statistically significant). There was no association between the use of PCSK9-mAb and an increased risk of neurocognitive adverse events (AEs), elevations of liver enzymes, rhabdomyolysis, or new-onset diabetes mellitus. The lack of effect on mortality may be explained by the short duration of the two cardiovascular outcome trials with evolocumab and alirocumab, FOURIER [4] and ODYSSEY OUTCOMES [5], which were completed rather fast, just after 2.2 and 2.8 years of follow-up, respectively. Of note, in the 4S [28] and LIPID [29] trials, the two statin trials which showed a decrease in all-cause- and CHD mortality, respectively, the event curves just started to diverge after about 1.5 years [30].

The effect of PCSK9-mAb on the aggregate of acute events across vascular territories has not been investigated. A post hoc analysis from the FOURIER study examined the effects of evolocumab on acute arterial events in all vascular territories (coronary, cerebrovascular, or peripheral vascular) and showed that they were all significantly decreased [31•]. There was a 19% reduction in first acute arterial events, a 17% reduction in acute coronary events, a 23% reduction in cerebrovascular, and a 42% reduction in peripheral events. The event reduction increased, as expected, over time (16% reduction in the first year and 24% reduction thereafter). There was a 35% reduction in recurrent events. The reduction in the incidence of events under evolocumab treatment emerged at about 9 months of therapy.

It seems that the significant beneficial atheroprotective effects of PCSK9-mAb need some time to fully develop.

### Plaque Composition

The effect of the PCSK9-mAb added to statin therapy on plaque burden and composition has till recently remained largely unknown. Two studies, one with evolocumab (the HUYGENS trial) [32•] and one with alirocumab (the PACMAN-AMI) [33•], contributed to answering this question. High risk characteristics of plaque vulnerability include a thin fibrous cap, a large lipid core, and a higher lipid pool [34]. In the double-blind placebo-controlled trial HUYGENS, 161 patients with a non-ST elevation MI (NSTEMI) were treated with evolocumab 420 mg per month (*n* = 80) or placebo (*n* = 81) for 52 weeks [32•]. Serial optical coherence tomography (OCT), reflecting plaque surface and individual components of the atheroma, and intravascular ultrasound (IVUS) imaging were performed at baseline and at week 50. The primary endpoint was the change in the minimum fibrous cap thickness (FCT) and maximum lipid arc in the imaged arterial segment. A total of 135 patients completed the study. More than 90% of the patients were receiving statin treatment with a high percentage of high-intensity statins in both groups. The first dose of evolocumab was given within 7 days of baseline imaging (OCT, IVUS) of a non-infarct related artery (non-IRA), following coronary angiography. The evolocumab group achieved lower LDL-C levels (28.1 vs. 87.2 mg/dl), a greater increase in minimum FCT (42.7 vs. 21.5 µm), and a decrease in maximum lipid arc. There was a greater regression of percent atheroma volume (PAT) seen with evolocumab compared with placebo (–2.29 vs. –0.61%), supporting the concept of early, aggressive lipid lowering after an acute MI.

The PACMAN-AMI study examined whether in patients with acute MI, either STEMI or NSTEMI, the addition of alirocumab on top of high-intensity statin therapy affects coronary atherosclerosis in non-IRAs [33•]. This was a randomized clinical trial including 300 patients who either received 150 mg alirocumab SC every 2 weeks or placebo, added to high-intensity statin therapy for 52 weeks. The first dose of alirocumab was given within 24 h after an urgent percutaneous intervention (PCI) of the culprit lesion in the IRA. There was a significantly greater reduction, more than double, in the mean change in PAT in non-IRAs as determined by IVUS (2.13% *vs.* 0.92%) in the alirocumab vs. placebo group, respectively.

The alirocumab group achieved lower LDL-C levels (23.8 vs. 84.4 mg/dl) and a greater increase in minimum FCT as determined by OCT (62.67 vs. 22.19 µm). Furthermore, there was a significant decrease in lipid core volume, more than double, based on a maximum lipid core burden as determined by near infrared spectroscopy (NIRS) (–79.42 with alirocumab vs. –37.60 with placebo).

These 2 studies strongly support a very intensive and early LDL-C lowering with evolocumab or alirocumab since it is associated with coronary plaque regression, lipid core reduction, and plaque stabilization.

### Lipoprotein(a)

Schwartz et al. [35•] examined in a post hoc analysis of the ODYSSEY OUTCOMES trial [5] the benefit of adding alirocumab in patients with LDL-C levels around 70 mg/dl, i.e., nominally controlled, and optimized statin treatment and if it is modified by the levels of Lp(a). They found that such patients with recent acute coronary syndrome had a significantly reduced risk for MACE when treated with alirocumab if they had just mildly elevated Lp(a) levels, higher than the median (≥ 13.7 mg/dl). However, the same patients showed no reduction in MACE if they had low Lp(a) levels, defined as < 13.7 mg/dl. These findings suggest that PCSK9 inhibition provides incremental clinical benefit in patients with LDL-C levels near 70 mg/dl only when the Lp(a) levels are at least mildly elevated. Of note, all post hoc analyses are primarily hypothesis-generating and require further validation.

The mechanism through which PCSK9-mAb decrease Lp(a) levels has not been clarified as of yet. Recently, researchers from Australia addressed this question [36•]. The effect of a 12-week alirocumab treatment (150 mg every 2 weeks) in 21 patients was compared in statin-treated patients with high and very high Lp(a) concentrations (median plasma concentrations at baseline ~ 50 mg/dl and ~ 100 mg/dl, respectively). It was shown that alirocumab decreases Lp(a) in the former group by increasing the fractional catabolic rate (FCR), i.e., the clearance of Lp(a), and in the latter by both, increasing clearance and decreasing the production rate of Lp(a) particles. Even though the results suggesting a dual mechanism of action for alirocumab are quite interesting, the size of the trial was rather small so that the results should be considered exploratory and requiring confirmation.

### Venous Thromboembolism

The relationship between cholesterol levels and the risk of venous thromboembolism (VTE) remains uncertain. Observational studies have yielded conflicting results [37, 38], while a Mendelian randomization study showed that persons with genetic predisposition for elevated LDL-C levels have a significantly increased risk for developing VTE [39]. The effect of PCSK9-mAb on the incidence of VTE was examined in a post hoc analysis of the FOURIER study [40]. The authors found a 46% reduction in the risk of VTE after 1 year of treatment with evolocumab, but not earlier. They also performed a meta-analysis of the FOURIER and ODYSSEY OUTCOMES trials and found a 31% relative risk reduction (RRR) in VTE associated with the use of PCSK9-mAb. Interestingly, there was a significant interaction between baseline Lp(a) concentrations and the degree of VTE risk reduction, with patients having an Lp(a) above the median of 37 nmol/l (~ 13 mg/dl) showing a reduction in risk of 48% and those with < 37 nmol/l showing no decrease in risk.

This was the first study to show a significant decrease in VTE with PCSK9-mAb. Since the reduction in VTE was associated with the degree of Lp(a)- but not LDL-C-lowering, it suggests that Lp(a) may be the mediator of VTE risk.

### Heart Failure

Since statins have not been shown to benefit patients with heart failure (HF) [41, 42], White et al. performed a post hoc analysis, examining the cardiovascular endpoints in ODYSSEY OUTCOMES in patients with or without a history of HF randomized to alirocumab or placebo [43•]. In total, 2815 (14.9%) patients had such a history. LDL-C reduction was achieved to the same degree in patients with or without HF. However, while alirocumab reduced the primary clinical endpoint (the composite of death from CHD, non-fatal MI, non-fatal or fatal ischemic stroke, or hospitalization for unstable angina) compared with placebo by 15% overall, this beneficial effect was observed only in patients without a history of HF (HR 0.78, 95% CI 0.70–0.86), but not in those with (HR 1.17, 95% CI 0.97–1.40). Further, alirocumab did not reduce hospitalization rates for HF, a pre-specified secondary outcome, overall and in the subgroup of patients with or without HF. Therefore, it seems that patients with HF may not benefit from PCSK9 inhibition and from intensive LDL-C lowering. However, as mentioned previously, results of subgroup analyses are only hypothesis-generating. The EVO-HF Pilot, an ongoing study expected to be completed mid-2022, will examine the effect of evolocumab in 46 patients with stable HF of ischemic etiology and with reduced ejection fraction (< 40%) [44]. A large prospective placebo-controlled evaluation of PCSK9 inhibition in patients with HF is needed in order to answer the question whether PCSK9-mAb are of benefit in patients with HF.

### Hyperglycemia

A number of Mendelian randomization studies [45, 46] and a meta-analysis [47] have suggested a potential association between PCSK9 inhibition and increased risk of diabetes mellitus. A recent study investigated this potential association using the FDA adverse event reporting system (FARS) (data from 7,295,624 eligible patients with 71,748 reports of evolocumab and 15,976 of alirocumab) [48]. It was shown that treatment with PCSK9-mAb was associated with increased reporting of hyperglycemic AEs (adjusted reporting odds ratio [adj. ROR] 1.14 [1.07–1.22]). They consisted mainly of mild hyperglycemia, but not diabetes. Hyperglycemic AEs were reported more frequently in patients with pre-existing diabetes, occurred mostly within the first 6 months of treatment, were reversible, and were reported less often when compared to statins. Interestingly, evolocumab but not alirocumab was associated with hyperglycemic AEs, a potential difference that needs to be investigated in prospective studies.

These findings strengthen the importance of early glucose monitoring for patients that start treatment with PCSK9-mAb, especially those with diabetes. Overall, the glycemic safety profile of PCSK9-mAb compared to statins is favorable. However, while findings of post-marketing surveillance programs can provide essential information, clinical trials are needed to further investigate this potential association, especially pharmacovigilance studies, which have played an important role in identifying side effects of other newly approved medications [49].

### Neurocognitive Function

Addressing the question whether PCSK9-mAb or the low LDL-C levels they are associated with are affecting cognition, a randomized trial in 1204 patients from the FOURIER trial followed for 19 months, the EBBINGHAUS trial, found no differences compared to placebo [50]. However, this trial included just < 5% of patients enrolled in the main FOURIER trial. Gencer et al. recently evaluated patient-reported cognition in a much larger cohort, the whole FOURIER trial population of 22,655 patients [51•]. They showed that evolocumab (always in addition to statins) had no impact on patient-reported cognition after an average of 2.2 years of treatment. This was true also for patients who reached an LDL-C of < 20 mg/dl. The absence of AEs on cognition was also shown with alirocumab (75 mg or 150 mg every 2 weeks) in a prospective, randomized trial of 2086 patients with hypercholesterolemia and at high or very high risk for cardiovascular events on maximally tolerated statin therapy [52]. They were assessed using the Cambridge Neuropsychological Test Automated Battery (CANTAB) cognitive domain Spatial Working Memory Strategy (SWMS) over 96 weeks of treatment. About 45% of patients on alirocumab achieved an LDL-C level of < 50 mg/dl. No effect on cognitive function with alirocumab was observed, irrespective of LDL-C levels.

Till now, the results on cognition and use of PCSK9-mAb and low LDL-C levels appear reassuring.

## Discussion

In the last couple of years, there has been an increasing number of approved nucleic acid–based therapeutics for the treatment of dyslipidemias. Antisense oligonucleotides such as volanesorsen targeting apolipoprotein C-III was approved in 2019 in Europe for the treatment of familial chylomicronemia and the siRNA inclisiran in 2020 in Europe and in 2021 in the USA for the treatment of hypercholesterolemia. Promising ASO for the treatment of hypertriglyceridemia such as olezarsen and for lowering Lp(a) such as pelacarsen are in advanced stages of development, as well as siRNAs such as olpasiran and SLN360 also targeting apolipoprotein(a) and decreasing Lp(a).

Although these treatments have elegant mechanisms of action which end up to the same result — the reduction of protein production — they have currently no data from cardiovascular outcome trials (CVOTs) providing evidence to support that they not only decrease pro-atherogenic lipoproteins but also ultimately reduce cardiovascular outcomes (or in the case of volanesorsen, the incidence of pancreatitis). A CVOT examining the effect of one of these therapies, namely, of the siRNA inclisiran, ORION-4, is currently recruiting and expected to be completed in 2026 [18].

In vivo editing of *PCSK9* durably decreases LDL-C levels in primates. However, potential off-target effects and the life-long effects of a factually irreversible treatment (re-editing back to the original genome would be associated with additional risks of off-target editing) need to be considered. There are further concerns. *PCSK9* knockout (KO) mice develop severe hepatic steatosis when fed a high-fat diet [53, 54], since PCSK9 reduces the expression of fatty acid translocase also known as CD36, a scavenger receptor for fatty acid uptake and the main driver of their uptake in the liver. PCSK9 expression, on the other hand, protects mice from diet-induced liver injury. Of note, it has been recently shown that treatment with the PCSK9-mAb alirocumab attenuated alcohol-induced steatohepatitis in a rat model [55]. Interestingly, while PCSK9-mAb decrease free-circulating PCSK9 [56–58], the levels of total plasma PCSK9 are increased [56, 59–61], most likely reflecting the longer half-life of the inactive PCSK9-mAb:PCSK9 complex [56, 59] and possibly also the compensatory increase in hepatic PCSK9 production [62].

Furthermore, a recent study in *PCSK9* KO mice [63] showed that PCSK9 deficiency, of most likely locally produced (in the heart muscle), not circulating, PCSK9 rewires heart metabolism and contributes to the development of HF with preserved ejection fraction — a puzzling observation, especially since PCSK9 is expressed at extremely low levels in mouse hearts [64].

Studies in humans with *PCSK9* editing tools have not started yet. It has to be considered that the maximal reductions in LDL-C levels of ~ 60% achieved till now with these methods match the ones achieved by approved medications such as statins and PCSK9-mAb. While the argument that *PCSK9* editing is a “once-and-done” approach that would guarantee by its nature life-long efficacy and compliance is valid, at the same time we would be introducing irreversible genomic changes for relatively modest returns. Of note, the genomic changes introduced involve only somatic, not germline cells, so they affect only the person treated and are not inherited.

In summary, at present and in the near future, PCSK9-mAb remain the only medications that robustly inhibit PCSK9, lower LDL-C, and reduce cardiovascular events with a satisfactory safety profile. If the siRNA inclisiran proves to be safe and effective in the ongoing CVOT ORION-4 trial, another therapeutic option to effectively fight ASCVD will be available. The ASO targeting PCSK9 seem also promising, albeit at relatively early (phase 2) stages of development. Whether gene-editing tools will be used in the clinic to lower LDL-C and cardiovascular disease risk remains to be determined when more data on the long-term safety of this are available. Of note, the companies involved in the AAV-meganuclease and LNP-ABE8.8 studies have both announced their plans to apply for regulatory approval to begin phase 1 clinical trials for patients with familial hypercholesterolemia (FH) in 2022 [65].

## Conclusions

While new promising methods of *PCSK9* inhibition are in development, PCSK9-mAb remain the drugs with the longest safety data available and the only ones having positive cardiovascular outcome trials. Regarding nucleic acid–based therapies, the siRNA targeting *PCSK9*, inclisiran, has already been approved for clinical use and its CVOT is underway. The ASO targeting *PCSK9* also seems promising but is still in early phases of clinical development. Editing of *PCSK9* is still in preclinical development with safety and efficacy issues in humans to be clarified. Due to of its permanent effects, long-term efficacy and safety data are needed before any clinical application is considered.

## References

[1] Virani SS, Alonso A, Aparicio HJ, Benjamin EJ, Bittencourt MS, Callaway CW (2021). Heart Disease and Stroke Statistics-2021 update: a report from the American Heart Association. Circulation.

[2] Townsend N, Wilson L, Bhatnagar P, Wickramasinghe K, Rayner M, Nichols M (2016). Cardiovascular disease in Europe: epidemiological update 2016. Eur Heart J.

[3] Shimada YJ, Cannon CP (2015). PCSK9 (Proprotein convertase subtilisin/kexin type 9) inhibitors: past, present, and the future. Eur Heart J.

[4] Sabatine MS, Giugliano RP, Keech AC, Honarpour N, Wiviott SD, Murphy SA (2017). Evolocumab and clinical outcomes in patients with cardiovascular disease. N Engl J Med.

[5] Schwartz GG, Steg PG, Szarek M, Bhatt DL, Bittner VA, Diaz R (2018). Alirocumab and cardiovascular outcomes after acute coronary syndrome. N Engl J Med.

[6] Kulkarni JA, Witzigmann D, Thomson SB, Chen S, Leavitt BR, Cullis PR (2021). The current landscape of nucleic acid therapeutics. Nat Nanotechnol.

[7] Warden BA, Duell PB (2021). Inclisiran: a novel agent for lowering apolipoprotein B-containing lipoproteins. J Cardiovasc Pharmacol..

[8] Katzmann JL, Packard CJ, Chapman MJ, Katzmann I, Laufs U (2020). Targeting RNA with antisense oligonucleotides and small interfering RNA: JACC state-of-the-art review. J Am Coll Cardiol.

[9] Landmesser U, Poller W, Tsimikas S, Most P, Paneni F, Luscher TF (2020). From traditional pharmacological towards nucleic acid-based therapies for cardiovascular diseases. Eur Heart J..

[10] Arsenault BJ (2022). The promise and challenges of RNA-targeted therapeutics in preventive cardiology. Eur Heart J.

[11] Musunuru K, Chadwick AC, Mizoguchi T, Garcia SP, DeNizio JE, Reiss CW (2021). In vivo CRISPR base editing of PCSK9 durably lowers cholesterol in primates. Nature.

[12] Rothgangl T, Dennis MK, Lin PJC, Oka R, Witzigmann D, Villiger L (2021). In vivo adenine base editing of PCSK9 in macaques reduces LDL cholesterol levels. Nat Biotechnol.

[13] Banerjee Y, Pantea Stoian A, Cicero AFG, Fogacci F, Nikolic D, Sachinidis A (2022). Inclisiran: a small interfering RNA strategy targeting PCSK9 to treat hypercholesterolemia. Expert Opin Drug Saf.

[14] Scicchitano P, Milo M, Mallamaci R, De Palo M, Caldarola P, Massari F (2021). Inclisiran in lipid management: a Literature overview and future perspectives. Biomed Pharmacother.

[15] Wright RS, Ray KK, Raal FJ, Kallend DG, Jaros M, Koenig W (2021). Pooled patient-level analysis of inclisiran trials in patients with familial hypercholesterolemia or atherosclerosis. J Am Coll Cardiol.

[16] Ray KK, Wright RS, Kallend D, Koenig W, Leiter LA, Raal FJ (2020). Two phase 3 trials of inclisiran in patients with elevated LDL cholesterol. N Engl J Med.

[17] Raal FJ, Kallend D, Ray KK, Turner T, Koenig W, Wright RS (2020). Inclisiran for the treatment of heterozygous familial hypercholesterolemia. N Engl J Med.

[18] Clinicaltrials.gov: A randomized trial assessing the effects of inclisiran on clinical outcomes among people with cardiovascular disease (ORION-4). https://www.clinicaltrials.gov/ct2/show/NCT03705234. Accessed 21 May 2022.

[19] •• Gennemark P, Walter K, Clemmensen N, Rekic D, Nilsson CAM, Knochel J, et al. An oral antisense oligonucleotide for PCSK9 inhibition. Sci Transl Med. 2021;13(593). 10.1126/scitranslmed.abe9117. **The first report on a potential oral ASO inhibiting PCSK9**. 10.1126/scitranslmed.abe9117 33980578

[20] •• Katzmann JL, Cupido AJ, Laufs U. Gene therapy targeting PCSK9. Metabolites. 2022;12(1). 10.3390/metabo12010070. **A comprehensive review on gene therapies targeting PCSK9**. 10.3390/metabo12010070 PMC8781734 35050192

[21] Wang L, Smith J, Breton C, Clark P, Zhang J, Ying L (2018). Meganuclease targeting of PCSK9 in macaque liver leads to stable reduction in serum cholesterol. Nat Biotechnol.

[22] Wang L, Breton C, Warzecha CC, Bell P, Yan H, He Z (2021). Long-term stable reduction of low-density lipoprotein in nonhuman primates following in vivo genome editing of PCSK9. Mol Ther.

[23] Breton C, Furmanak T, Avitto AN, Smith MK, Latshaw C, Yan H (2021). Increasing the specificity of AAV-based gene editing through self-targeting and short-promoter strategies. Mol Ther.

[24] Nishiga M, Liu C, Qi LS, Wu JC (2022). The use of new CRISPR tools in cardiovascular research and medicine. Nat Rev Cardiol.

[25] Akinc A, Querbes W, De S, Qin J, Frank-Kamenetsky M, Jayaprakash KN (2010). Targeted delivery of RNAi therapeutics with endogenous and exogenous ligand-based mechanisms. Mol Ther.

[26] van Kampen SJ, van Rooij E (2021). CRISPR base editing lowers cholesterol in monkeys. Nat Biotechnol.

[27] • Guedeney P, Giustino G, Sorrentino S, Claessen BE, Camaj A, Kalkman DN, et al. Efficacy and safety of alirocumab and evolocumab: a systematic review and meta-analysis of randomized controlled trials. Eur Heart J. 2019. 10.1093/eurheartj/ehz430. **Large meta-analysis using data from 39 phase 2 or phase 3 randomized controlled trials and totalling 66,478 patients showing the efficacy and safety of evolocumab and alirocumab**. 10.1093/eurheartj/ehz430 31270529

[28] Randomised trial of cholesterol lowering in 4444 patients with coronary heart disease: the Scandinavian Simvastatin Survival Study (4S). Lancet. 1994;344(8934):1383–9. 7968073

[29] Long-Term Intervention with Pravastatin in Ischaemic Disease Study G. Prevention of cardiovascular events and death with pravastatin in patients with coronary heart disease and a broad range of initial cholesterol levels. N Engl J Med. 1998;339(19):1349–57. 10.1056/NEJM199811053391902. 10.1056/NEJM199811053391902 9841303

[30] Sabatine MS (2019). PCSK9 inhibitors: what we know, what we should have understood, and what is to come. Eur Heart J.

[31] Oyama K, Giugliano RP, Tang M, Bonaca MP, Saver JL, Murphy SA (2021). Effect of evolocumab on acute arterial events across all vascular territories : results from the FOURIER trial. Eur Heart J..

[32] Nicholls SJ, Kataoka Y, Nissen SE, Prati F, Windecker S, Puri R (2022). Effect of evolocumab on coronary plaque phenotype and burden in statin-treated patients following myocardial infarction. JACC Cardiovasc Imaging..

[33] Raber L, Ueki Y, Otsuka T, Losdat S, Haner JD, Lonborg J (2022). Effect of alirocumab added to high-intensity statin therapy on coronary atherosclerosis in patients with acute myocardial infarction: the PACMAN-AMI Randomized Clinical Trial. JAMA..

[34] Prati F, Romagnoli E, Gatto L, La Manna A, Burzotta F, Ozaki Y (2020). Relationship between coronary plaque morphology of the left anterior descending artery and 12 months clinical outcome: the CLIMA study. Eur Heart J.

[35] Schwartz GG, Szarek M, Bittner VA, Diaz R, Goodman SG, Jukema JW (2021). Lipoprotein(a) and benefit of PCSK9 inhibition in patients with nominally controlled LDL cholesterol. J Am Coll Cardiol..

[36] Ying Q, Chan DC, Pang J, Marcovina SM, Barrett PHR, Watts GF (2022). PCSK9 inhibition with alirocumab decreases plasma lipoprotein(a) concentration by a dual mechanism of action in statin-treated patients with very high apolipoprotein(a) concentration. J Intern Med..

[37] Kunutsor SK, Seidu S, Khunti K (2017). Statins and secondary prevention of venous thromboembolism: pooled analysis of published observational cohort studies. Eur Heart J.

[38] van Schouwenburg IM, Mahmoodi BK, Gansevoort RT, Muntinghe FL, Dullaart RP, Kluin-Nelemans HC (2012). Lipid levels do not influence the risk of venous thromboembolism. Results of a population-based cohort study. Thromb Haemost.

[39] Klarin D, Busenkell E, Judy R, Lynch J, Levin M, Haessler J (2019). Genome-wide association analysis of venous thromboembolism identifies new risk loci and genetic overlap with arterial vascular disease. Nat Genet.

[40] Marston NA, Gurmu Y, Melloni GEM, Bonaca M, Gencer B, Sever PS (2020). The effect of PCSK9 (proprotein convertase subtilisin/kexin type 9) inhibition on the risk of venous thromboembolism. Circulation.

[41] Tavazzi L, Maggioni AP, Marchioli R, Barlera S, Franzosi MG, Latini R (2008). Effect of rosuvastatin in patients with chronic heart failure (the GISSI-HF trial): a randomised, double-blind, placebo-controlled trial. Lancet.

[42] Kjekshus J, Apetrei E, Barrios V, Bohm M, Cleland JG, Cornel JH (2007). Rosuvastatin in older patients with systolic heart failure. N Engl J Med.

[43] White HD, Schwartz GG, Szarek M, Bhatt DL, Bittner VA, Chiang CE (2022). Alirocumab after acute coronary syndrome in patients with a history of heart failure. Eur Heart J.

[44] Clinicaltrials.gov: EVOlocumab in stable heart failure with reduced ejection fraction of ischemic etiology: EVO-HF Pilot. https://clinicaltrials.gov/ct2/show/NCT03791593. Accessed 21 May 2022.

[45] Schmidt AF, Swerdlow DI, Holmes MV, Patel RS, Fairhurst-Hunter Z, Lyall DM (2017). PCSK9 genetic variants and risk of type 2 diabetes: a mendelian randomisation study. Lancet Diabetes Endocrinol.

[46] Ference BA, Robinson JG, Brook RD, Catapano AL, Chapman MJ, Neff DR (2016). Variation in PCSK9 and HMGCR and risk of cardiovascular disease and diabetes. N Engl J Med.

[47] Lotta LA, Sharp SJ, Burgess S, Perry JRB, Stewart ID, Willems SM (2016). Association between low-density lipoprotein cholesterol-lowering genetic variants and risk of type 2 diabetes: a meta-analysis. JAMA.

[48] Goldman A, Raschi E, Cukierman-Yaffe T, Dankner R, Shouval R, Shechter M (2021). Hyperglycaemic disorders associated with PCSK9 inhibitors: a real-world, pharmacovigilance study. Eur J Prev Cardiol.

[49] Raschi E, Poluzzi E, Salvo F, Pariente A, De Ponti F, Marchesini G (2018). Pharmacovigilance of sodium-glucose co-transporter-2 inhibitors: what a clinician should know on disproportionality analysis of spontaneous reporting systems. Nutr Metab Cardiovasc Dis.

[50] Giugliano RP, Mach F, Zavitz K, Kurtz C, Im K, Kanevsky E (2017). Cognitive function in a randomized trial of evolocumab. N Engl J Med.

[51] Gencer B, Mach F, Guo J, Im K, Ruzza A, Wang H (2020). Cognition after lowering LDL-cholesterol with evolocumab. J Am Coll Cardiol..

[52] Janik MJ, Urbach DV, van Nieuwenhuizen E, Zhao J, Yellin O, Baccara-Dinet MT (2021). Alirocumab treatment and neurocognitive function according to the CANTAB scale in patients at increased cardiovascular risk: a prospective, randomized, placebo-controlled study. Atherosclerosis.

[53] Ioannou GN, Lee SP, Linsley PS, Gersuk V, Yeh MM, Chen YY (2022). Pcsk9 deletion promotes murine nonalcoholic steatohepatitis and hepatic carcinogenesis: role of cholesterol. Hepatol Commun.

[54] Lebeau PF, Byun JH, Platko K, Al-Hashimi AA, Lhotak S, MacDonald ME (2019). Pcsk9 knockout exacerbates diet-induced non-alcoholic steatohepatitis, fibrosis and liver injury in mice. JHEP Rep.

[55] Lee JS, Mukhopadhyay P, Matyas C, Trojnar E, Paloczi J, Yang YR (2019). PCSK9 inhibition as a novel therapeutic target for alcoholic liver disease. Sci Rep.

[56] Roth EM, Kastelein JJP, Cannon CP, Farnier M, McKenney JM, DiCioccio AT (2020). Pharmacodynamic relationship between PCSK9, alirocumab, and LDL-C lowering in the ODYSSEY CHOICE I trial. J Clin Lipidol.

[57] Stein EA, Mellis S, Yancopoulos GD, Stahl N, Logan D, Smith WB (2012). Effect of a monoclonal antibody to PCSK9 on LDL cholesterol. N Engl J Med.

[58] Desai NR, Giugliano RP, Wasserman SM, Gibbs JP, Liu T, Scott R (2017). Association between circulating baseline proprotein convertase subtilisin kexin type 9 levels and efficacy of evolocumab. JAMA Cardiol.

[59] Shapiro MD, Miles J, Tavori H, Fazio S (2018). Diagnosing resistance to a proprotein convertase subtilisin/kexin type 9 inhibitor. Ann Intern Med.

[60] Rey J, Poitiers F, Paehler T, Brunet A, DiCioccio AT, Cannon CP, et al. Relationship between low-density lipoprotein cholesterol, free proprotein convertase subtilisin/kexin type 9, and alirocumab levels after different lipid-lowering strategies. J Am Heart Assoc. 2016;5(6). 10.1161/JAHA.116.003323. 10.1161/JAHA.116.003323 PMC4937273 27287699

[61] Shapiro MD, Tavori H, Fazio S (2018). PCSK9: from basic science discoveries to clinical trials. Circ Res.

[62] Oleaga C, Shapiro MD, Hay J, Mueller PA, Miles J, Huang C (2021). Hepatic sensing loop regulates PCSK9 secretion in response to inhibitory antibodies. J Am Coll Cardiol.

[63] Da Dalt L, Castiglioni L, Baragetti A, Audano M, Svecla M, Bonacina F (2021). PCSK9 deficiency rewires heart metabolism and drives heart failure with preserved ejection fraction. Eur Heart J.

[64] Zaid A, Roubtsova A, Essalmani R, Marcinkiewicz J, Chamberland A, Hamelin J (2008). Proprotein convertase subtilisin/kexin type 9 (PCSK9): hepatocyte-specific low-density lipoprotein receptor degradation and critical role in mouse liver regeneration. Hepatology.

[65] Whittaker MN, Musunuru K (2022). Therapeutic application of genome editing in dyslipidemia. Curr Opin Lipidol.

[66] Tsimikas S, Moriarty PM, Stroes ES (2021). Emerging RNA therapeutics to lower blood levels of Lp(a): JACC Focus Seminar 2/4. J Am Coll Cardiol.

[67] Katzmann JL, Gouni-Berthold I, Laufs U (2020). PCSK9 inhibition: insights from clinical trials and future prospects. Front Physiol.

[68] Hovingh GK, Lepor NE, Kallend D, Stoekenbroek RM, Wijngaard PLJ, Raal FJ (2020). Inclisiran durably lowers low-density lipoprotein cholesterol and proprotein convertase subtilisin/kexin type 9 expression in homozygous familial hypercholesterolemia: the ORION-2 Pilot Study. Circulation.

